# A Nomogram Predicting the Progression-Free Survival of Nonmetastatic Renal Cell Carcinoma Patients With Venous Thrombus After Surgery

**DOI:** 10.3389/fonc.2022.765092

**Published:** 2022-03-24

**Authors:** Yu Zhang, XiaoJun Tian, Hai Bi, Ye Yan, Zhuo Liu, Cheng Liu, ShuDong Zhang, LuLin Ma

**Affiliations:** Department of Urology, Peking University Third Hospital, Beijing, China

**Keywords:** RCC, venous thrombus, nomogram, PFS, RN-VT

## Abstract

**Objectives:**

To demonstrate the progression-free survival (PFS) of nonmetastatic renal cell carcinoma (RCC) patients with venous thrombus after radical nephrectomy and venous thrombectomy (RN-VT) and to develop and validate a nomogram to predict the PFS of patients after RN-VT.

**Materials and Methods:**

We reported our prospective follow-up data of RCC patients with venous thrombus from January 2014 to September 2020 (n = 199). We used the Kaplan–Meier method to assess the PFS. The Cox proportional hazards regression model was used to determine the predictors. Nomograms predicting the PFS was established, and external validation was performed. Calibration curves and decision curves were generated to assess the predictive efficacy and clinical benefit.

**Results:**

After a median follow-up of 32 months, 79 patients (39.7%) had disease progression and the median PFS was 41.0 months (95% CI 34.8–53.2 months). The 1-year, 3-year, and 5-year PFS rates were 78.4%, 45.4%, and 30.0%, respectively. Multivariate analysis showed that Fuhrman grade [grade 4: hazard ratio (HR) 1.92, 95% CI 1.10–3.34, P = 0.02], pathological type (papillary RCC: HR 3.02, 95% CI 1.79–5.10, P < 0.001), perinephric fat invasion (HR 1.54, 95% CI 1.12–2.10, P = 0.007), sarcomatoid differentiation (HR 2.97, 95% CI 1.24–7.13, P = 0.02) were associated with a worse PFS, and adjuvant therapy (HR 0.32, 95% CI 0.18–0.59, P < 0.001) could lead to a better PFS. A nomogram based on the predictors was externally validated to have good discrimination and calibration, and it could improve PFS prediction to obtain a clinical benefit.

**Conclusions:**

We constructed and validated a nomogram to predict the 1-year, 3-year, and 5-year PFS of M0 RCC patients with venous thrombus after surgery. The model can help identify patients who can benefit the most from surgery and develop the criteria for clinical trial enrollment.

## Introduction

Radical nephrectomy and venous thrombectomy (RN-VT) has been the curative option for renal cell carcinoma (RCC) patients with venous thrombus (1–3). Previous studies focusing on the survival of such patients reported that the 5-year cancer-specific survival could be 46.0%–53.4%, and the 5-year overall survival could be 39%–42.2% after RN-VT (2, 4–6). These findings supported the benefit of RN-VT in this patient group. However, the 3-year recurrence-free survival was reported to be only 35.9% for M0 patients with thrombus extending above the hepatic veins (6). This highlighted the high recurrence risk nature in such patients and reminded us of the disease progression during follow-up.

To date, no systematic effort has been made to assess the progression-free survival (PFS) of M0 RCC patients with venous thrombus after surgery. In the current study, we aimed to report the PFS based on the prospective follow-up data and to assess the prognostic factors in M0 RCC patients with venous thrombus. Besides that, we endeavored to develop and validate a model to better predict the PFS. We hypothesized that our nomogram could help predict individual PFS probability with good discrimination and calibration and lead to a clinical benefit.

## Materials and Methods

### Study Cohort and Design

We have been building the Peking University Third Hospital Thrombus Database (PUTH-TD) since January 2014 after obtaining the institutional review board approval. Our study cohort contained RCC patients with venous thrombus from January 2014 to September 2020, and all patients were prospectively followed up to March 2021. The inclusive criteria were as follows: (1) patients with pathologically confirmed RCC; (2) patients treated with surgical procedures. The patients with a minimum follow-up of less than 6 months and distant metastasis were excluded. A total of 199 patients were enrolled in the study. All procedures were performed by experienced surgeons. Adjuvant therapy is one of the comprehensive therapeutic methods of cancer, including cytokine therapy, radiotherapy, or targeted therapy. All the patients at our institution were recommended to receive adjuvant therapy unless the patients could not tolerate the toxicity of adjuvant therapy due to severe complications or poor postoperative physical condition.

### Outcomes and Definitions

The primary endpoint was the PFS after surgery. Progression was defined as local recurrence (tumor recurrence in or abutting the previous surgical bed), distant metastasis progression (new lesions in other organs, brain, lung, liver, bone, et al.), or death from tumor after surgery. PFS was defined as the time from surgery to the progression event. The local recurrence and distant metastasis were evaluated based on computed tomography (CT) or magnetic resonance imaging (MRI).

Local symptoms were defined as a palpable mass, pain, and gross hematuria. Patients with edema, fever, swelling, fatigue, and weight loss were thought to have systemic symptoms. The American Society of Anesthesiologists Physical Status classification system (ASA level) (7) was introduced to estimate the operative risk. Complications were graded according to the Clavien–Dindo grading system (8). Severe complication was defined as Clavien–Dindo grade above II.

We classified the thrombus into three levels: 1) level I, renal vein thrombus (Mayo 0); 2) level II, thrombus extending into the renal vein but below the intrahepatic vena cava (Mayo I and II); and 3) level III, thrombus extending into the intrahepatic vena cava or even into the right atrium (Mayo III and IV) (1, 9). The histological diagnosis of renal tumors was based on the World Health Organization (WHO) classification (2004 and 2016 version) (10, 11). The Fuhrman system was applied to RCC nuclear grading (12). A sarcomatoid differentiation was defined as RCC accompanied by histological appearance of spindle-cell sarcoma. The 2017 version of the tumor-node-metastasis (TNM) classification was used for clinical staging based on postoperative pathological specimens.

### Follow-Up Protocol

Two full-time clinical data managers had all access to the database and performed the follow-up. We provided the same follow-up plan to all patients, and follow-up data were prospectively collected (symptoms and signs, laboratory tests, imaging examination of the chest, abdomen, and pelvis). The laboratory tests included routine blood test and blood biochemical test. The imaging examination included CT, MRI, and X-ray. Patients were followed up every 3 months after surgery in the first year, then 6 months to the third year, then annually thereafter. Except for the routine review after surgery, the data managers conducted telephone interviews every 3–6 months and collected the follow-up information to reduce the withdraw bias.

### Nomogram Construction and External Validation

Nomograms were built based on the predictors determined by Cox proportional hazards regression analysis, and the C-index was calculated to assess the discrimination of the model. We calculated the total points of each patient in the validation cohort according to the nomogram established based on the training cohort. The total point in the validation cohort was used as a factor in the Cox regression analysis, and the C-index and calibration curve were derived according to the regression analysis. We randomly grouped patients in a ratio of 3:1 to determine the training cohort and validation cohort.

### Statistical Analysis

Baseline characteristics were shown for categorical variables and continuous variables. Non-normally distributed continuous variables were reported as medians and interquartile ranges, and normally distributed continuous variables were reported as means and standard deviations. We reported the categorical variables as frequencies and proportions. The chained multiple imputation was used to resolve the missing data.

We used the Kaplan–Meier method to perform PFS analysis. Cox proportional hazards regression analysis was used to estimate the predictors of PFS, which should satisfy the proportional hazards assumptions first. Calibration curves and decision curves were generated to assess the calibration and clinical benefit.

All statistical tests were performed by SPSS version 25.0 (IBM, Armonk, NY, USA) and the R statistics package version 4.1.0 (R Project for Statistical Computing, www.r-project.org). All tests were two-sided, and the significance level was set at P < 0.05.

## Results

### Baseline Characteristics and Outcomes of Study Cohort

Patient characteristics are listed in **Table 1**. A total of 199 patients with RCC and venous thrombus formed the study cohort, including 56 patients (28.1%) with level I thrombus, 109 patients (54.8%) with level II thrombus, and 34 patients (17.1%) with level III thrombus. Among them, 79 patients (39.7%) had progression events, including death (n = 44), local recurrence or distant metastasis progression (n = 35). Of the 199 patients, 167 patients (83.9%) had clear cell RCC, 25 patients (12.6%) had papillary RCC, and 7 patients (3.5%) had other RCC types. Lymph node metastasis was confirmed in 58 patients (29.1%), and perinephric fat invasion was found in 42 patients (21.1%). The venous wall was involved in 43 patients (21.6%), and 36 patients (18.1%) received segmental resection of Inferior vena cava (IVC). The number of Fuhrman grade I, II, III, and IV RCC patients was 4 (2.0%), 76 (38.2%), 89 (44.7%), and 30 (15.1%), respectively. A total of 103 patients (51.8%) received adjuvant therapy, 39 patients (19.6%) did not receive adjuvant therapy due to poor postoperative physical condition or severe complications, and 57 patients (28.6%) refused it due to high cost. The surgical, pathological, and oncologic outcomes were shown in **Table 2**.

**Table 1:** Baseline characteristics of the study cohort (n = 199).

Characteristics	Value
Age (years), median (IQR)	60 (54–67)
Gender (n/%)	
Male	153 (76.9)
Female	46 (23.1)
BMI (kg/m^2^), median (IQR)	24.2 (21.9–27.0)
Laterality (n/%)	
Left	82 (41.2)
Right	117 (58.8)
ASA level (n/%)	
1	9 (4.5)
2	166 (83.4)
3	23 (11.6)
4	1 (0.5)
Symptoms (n/%)	
Local	129 (76.9)
Systemic	70 (31.1)
Comorbidity (n/%)	
Hypertension	81 (40.7)
Coronary heart disease	11 (5.5)
Diabetes mellitus	26 (13.1)
Cerebrovascular disease	5 (2.5)
Surgery history	49 (24.6)
Preoperative targeted therapy (n/%)	9 (4.5)
Tumor diameter (cm), median (IQR)	8.2 (6.5–10.0)
Preoperative SCR (μmol/L), median (IQR)	90.8 (80.4–107)
Thrombus level (n/%)	
I	56 (28.1)
II	109 (54.8)
III	34 (17.1)
Pulmonary embolism (n/%)	2 (1.0)
Metastasis at diagnosis (n/%)	
Suspected lymph node metastasis	110 (55.3)
Suspected adrenal metastasis	9 (4.5)

BMI, body mass index; ASA, American Society of Anesthesiologists; SCR, serum creatine; IQR, interquartile range.

**Table 2:** Surgical, pathological, and oncologic outcomes of the study cohort.

Characteristics	Value
Adrenalectomy (n/%)	80 (40.2)
Segmental resection of IVC (n/%)	36 (18.1)
Operative time (min), median (IQR)	321 (239–409)
Blood loss (ml), median (IQR)	600 (200–1,525)
Blood transfusion (n/%)	90 (45.2)
Packed RBC transfusion (ml), median (IQR)	1,200 (800–2,000)
FFP transfusion (ml), median (IQR)	600 (500–800)
Postoperative SCR	96 (78–113)
Complications (n/%)	61 (30.7)
Clavien grade of complications (n/%)	
I	6 (3.0)
II	53 (26.6)
III	2 (1.0)
Postoperative hospital stay (days), median (IQR)	9 (6–13)
Histology (n/%)	
Clear cell RCC	167 (83.9)
Papillary type RCC	25 (12.6)
Other RCC	7 (3.5)
T stage	
pT3a	36 (18.1)
pT3b	94 (47.2)
pT3c	64 (32.2)
pT4	5 (2.5)
Lymph node metastasis	58 (29.1)
Perinephric fat invasion	42 (21.1)
Involving the venous wall (n/%)	43 (21.6)
Sarcomatoid differentiation	17 (8.5)
Fuhrman grade (n/%)	
1	4 (2.0)
2	76 (38.2)
3	89 (44.7)
4	30 (15.1)
Adjuvant therapy	103 (51.8)
Targeted therapy	100 (50.3)
Radiotherapy	3 (1.5)
Progression events (n/%)	79 (39.7)
Death	44 (22.1)
Recurrence or metastatic progression	35 (17.6)

RCC, renal cell carcinoma; IVT, inferior vena cava; RBC, red blood cells; FFP, fresh frozen plasma; ASA, American Society of Anesthesiologists; IQR, interquartile range; SCR, serum creatinine.

### Progression-Free Survival

**Figure 1:**
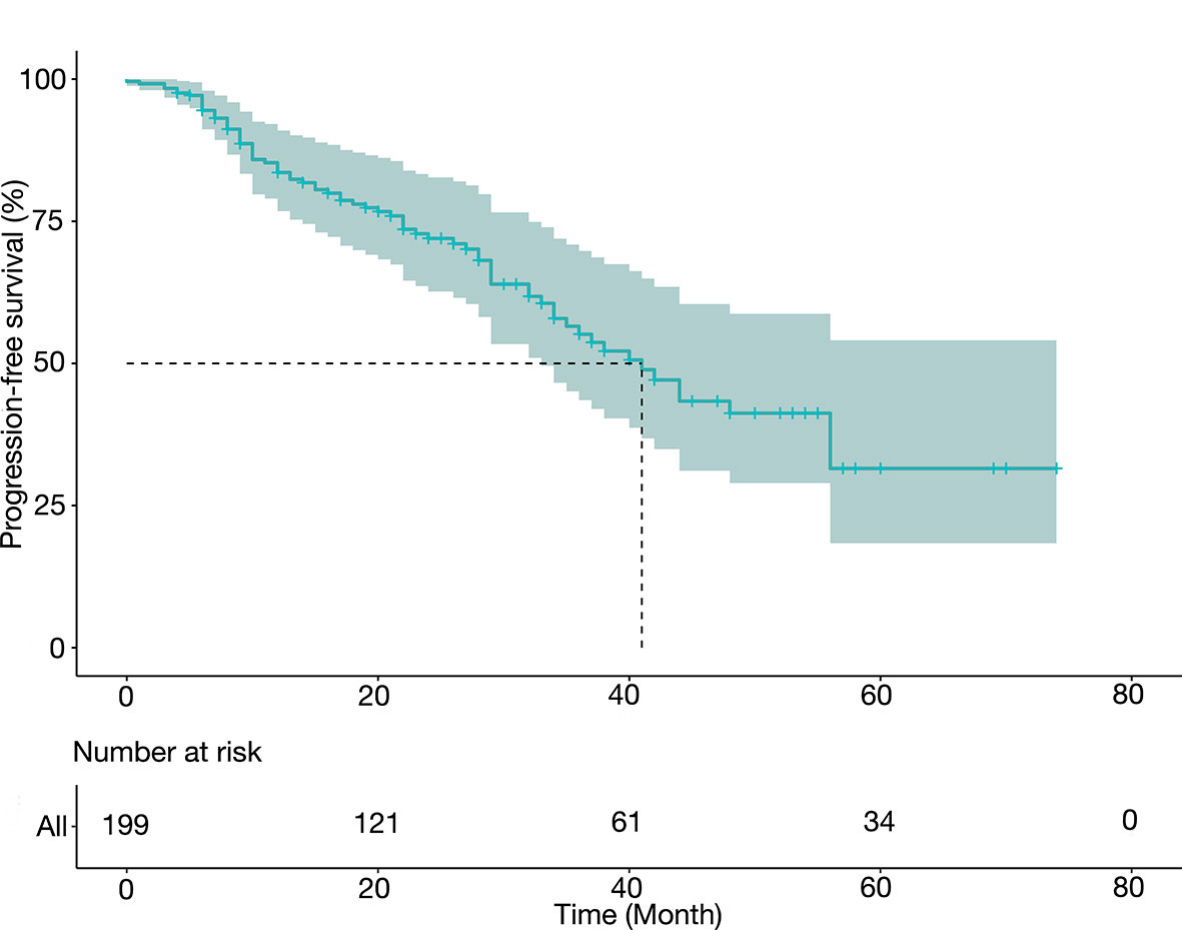
Adjusted progression-free survival (PFS) of the study cohort.

**Table 3** summarized the PFS data and presented them in groups. After a median follow-up of 32 months, the median PFS was 41.0 months (34.8–53.2 months), and the 1-year, 3-year, and 5-year PFS rates were 78.4%, 45.4%, and 30.0%, respectively. The median PFS was 44.3 months (95% CI 39.9–48.8 months) for level I thrombus, 41.6 months (95% CI 35.8–47.5 mon) for level II thrombus, and 38.1 months (95% CI 30.8–45.3 months) for level III thrombus. **Figure 1** depicted the PFS curve.

**Table 3:** Progression-free survival of the study cohort and subgroup analysis.

	PFS	1-year	3-year	5-year
	Median months, (95% CI)			
All	41.0 (34.8–53.2)	78.4%	45.4%	30.0%
N0-xM0	44.0 (35.1–52.9)	81.3%	51.7%	35.3%
N1M0	29.0 (24.7–33.2)	65.5%	40.6%	23.0%
Thrombus level				
I	44.3 (39.9–48.8)	82.3%	65.6%	49.1%
II	41.6 (35.8–47.5)	77.2%	51.5%	46.5%
III	38.1 (30.8–45.3)	75.0%	39.7%	24.8%

PFS, progression-free survival; CI, confidence interval.

### Univariate and Multivariate Analyses of Progression-Free Survival

Though some predictors were found in univariate analysis, multivariate analysis showed that only Fuhrman grade (grade 4, HR 1.92, 95% CI 1.10–3.34, P = 0.02), pathological type (papillary RCC, HR 3.02, 95% CI 1.79–5.10, P < 0.001), perinephric fat invasion (HR 1.54, 95% CI 1.12–2.10, P = 0.007), sarcomatoid differentiation (HR 2.97, 95% CI 1.24–7.13, P = 0.02), and adjuvant therapy were associated with PFS (**Table 4**).

**Table 4:** Univariate and multivariate Cox regression analysis.

Covariate	PFS			
	Univariate analysis	P value	Multivariate analysis	P value
	HR (95% CI)		HR (95% CI)	
Age	0.97 (0.96–0.99)	0.02	1.01 (0.99–1.04)	0.35
BMI	0.98 (0.94–1.02)	0.33	–	–
Thrombus level				
I	Reference	–	–	–
II	1.05 (0.66–1.68)	0.82	–	–
III	1.54 (0.91–2.59)	0.11	–	–
Severe complications				
No	Reference		Reference	
Yes	3.31 (1.94–5.65)	<0.001	1.96 (0.60–6.35)	0.26
Fuhrman grade				
1–2	Reference		Reference	
3	1.48 (1.10–1.99)	0.01	1.48 (0.92–2.37)	0.11
4	2.82 (2.03–3.92)	<0.001	1.92 (1.10–3.34)	0.02
Pathological type				
Clear cell RCC	Reference		Reference	
Papillary RCC	2.58 (1.58–4.21)	<0.001	3.02 (1.79–5.10)	<0.001
Other	2.17 (0.88–5.38)	0.09	0.89 (0.11–6.99)	0.91
Perinephric fat invasion				
No	Reference		Reference	
Yes	1.69 (1.15–2.50)	0.008	1.54 (1.12–2.10)	0.007
Sarcomatoid differentiation				
No	Reference		Reference	
Yes	3.33 (1.99–5.58)	<0.001	2.97 (1.24–7.13)	0.02
Lymph node metastasis				
N0M0	Reference		Reference	
N1-xM0	1.30 (0.90–1.88)	0.17	1.10 (0.70–1.74)	0.68
Adjuvant therapy				
No	Reference		Reference	
Yes	0.44 (0.24–0.79)	0.007	0.32 (0.18–0.59)	<0.001

PFS, progression-free survival; HR, hazard ratio; CI, confidence interval; BMI, body mass index; RCC, renal cell carcinoma.

### Development and External Validation of Nomogram Predicting Progression-Free Survival

**Figure 2:**
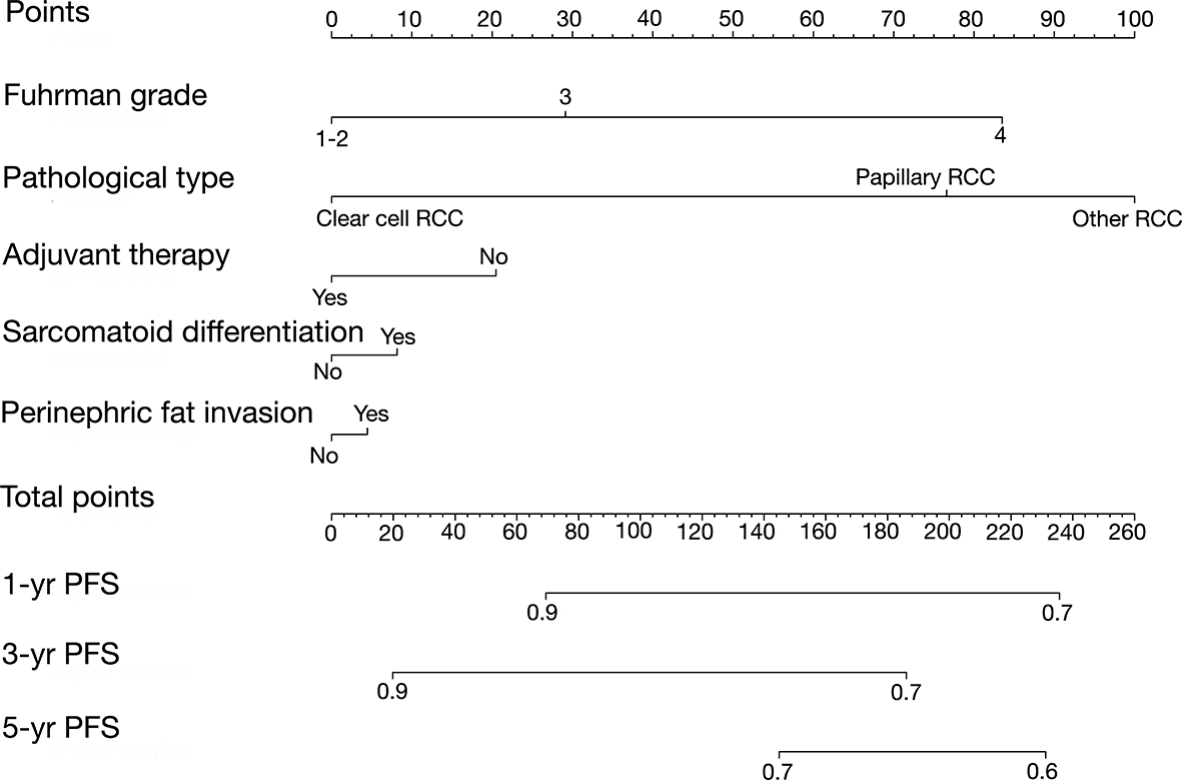
The Peking University nomogram (PKUN) model predicting 1-year, 3-year, and 5-year PFS.

For the construction of the PFS model, patients were split randomly into the training cohort (n = 148) and validation cohort (n = 51). We developed the Peking University nomogram (PKUN) based on the predictors identified in the multivariate analysis to predict the 1-year, 3-year, and 5-year PFS after RN-VT. The predictive C-indexes for 1-year, 3-year, and 5-year PFS were 0.83, 0.77, and 0.78 (**Figure 2**).

**Figure 3:**
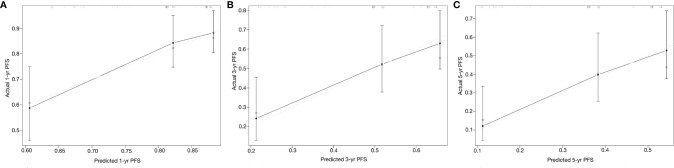
Calibration curves of the Peking University nomogram (PKUN) model. **(A)** for 1-year PFS, **(B)** for 3-year PFS, and **(C)** for 5-year PFS.

**Figure 4:**
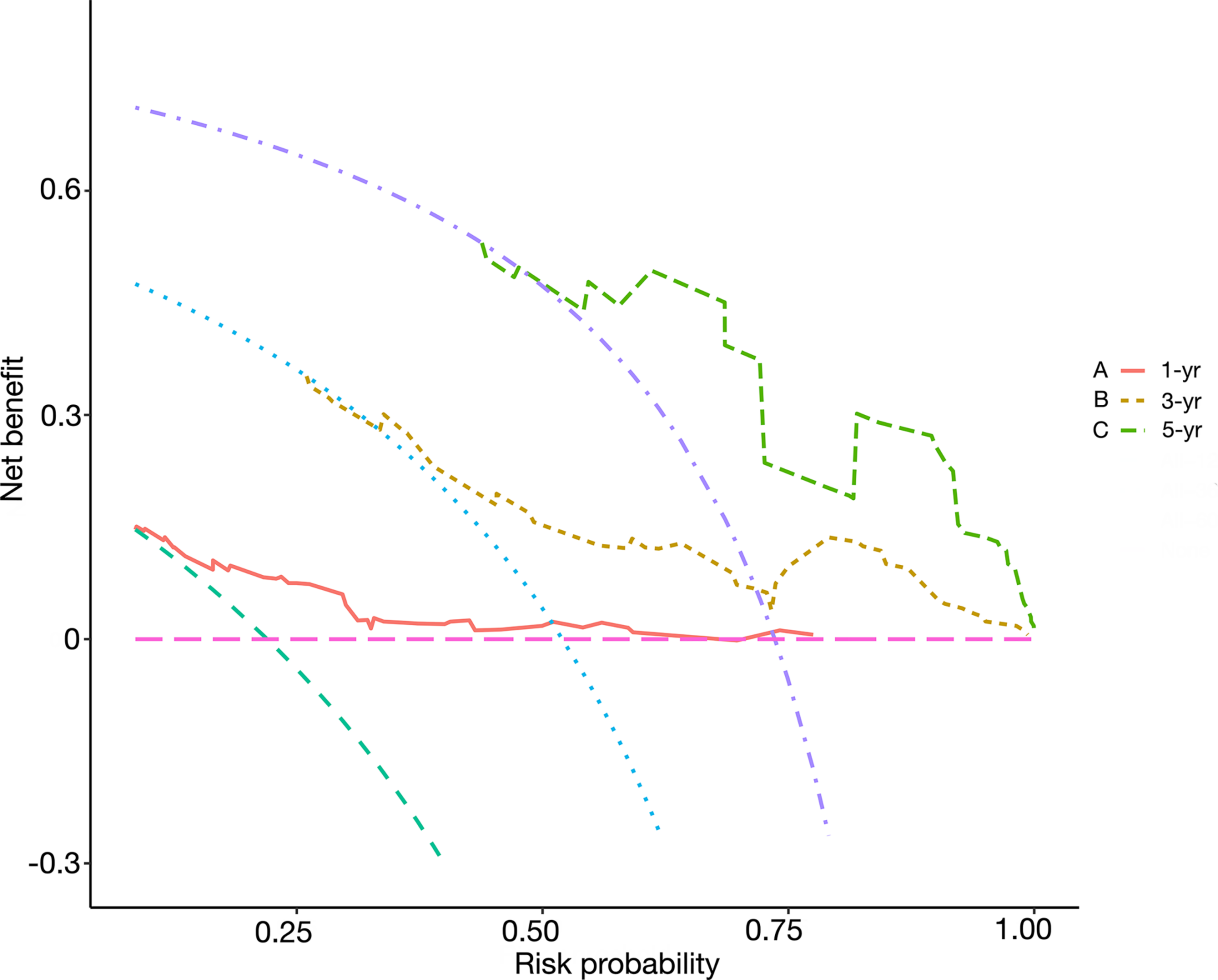
Decision curves of the Peking University nomogram (PKUN) model. **(A)** for 1-year PFS, **(B)** for 3-year PFS, and **(C)** for 5-year PFS.

On each calibration plot, the predicted PFS probability is represented on the x-axis, and the actual risk of progression is represented on the y-axis. The 45° line indicates perfect agreement between predicted probability and observed risk. The externally validated calibration plots demonstrated virtually overlapping the 45° line of progression (**Figures 3A–C**). The decision curve analysis demonstrated the net clinical benefit originating from applying this model with probabilities >1% for 1-year PFS, >25% for 3-year PFS, and >40% for 5-year PFS (**Figure 4**).

## Discussion

Progression of RCC patients with venous thrombus has been rarely evaluated, though the long-term survival was confirmed to be acceptable (2, 4, 6). Here, we reported that such M0 patients had a median PFS of 41 months, and that the 5-year PFS was only 30.0%. To provide an individualized PFS prediction, we developed the PKUN model and validated that the PKUN model had good discrimination and calibration. We anticipated that the PKUN model would be useful for patient counseling, treatment decision-making, and enrollment of clinical trials in the RCC and venous thrombus population.

Several studies evaluated the overall survival or cancer-specific survival of RCC patients with venous thrombus (4, 6, 13). However, the PFS was less reported. Reese et al. (14) once reported that the median PFS was only 5 months and the 1-year PFS was 29.0% in untreated RCC patients with venous thrombus. Rigaud et al. (2) reported that the 5-year PFS was 8.9% in 40 patients receiving surgery after a median follow-up of 28.5 months. In our study, the 5-year PFS was 30.0% for the entire cohort. The following reasons could explain the higher 5-year PFS at our institution. Firstly, the proportion of nuclear grade III and IV was much higher in their cohort (90% vs. 69.8%). Next, 47.5% of the patients in their study had perinephric fat invasion, higher than 21.1% of our cohort. Lastly, the proportion of patients receiving adjuvant therapy was 51.8% in our cohort. That might help reduce the risk of disease progression.

Individual prediction of PFS of patients after surgery is important to perform precise adjuvant therapy. To better evaluate the risk of progression, we developed and validated the PKUN model. Our nomogram could be used to inform RCC patients with venous thrombus the necessity of adjuvant therapy. Several aspects of our nomogram were noteworthy. First, our result showed that thrombus level was not a prognostic factor of PFS. Actually, the predictive value of thrombus level on long-term survival was still controversial (9, 15, 16). However, Abel et al. (17) reported that thrombus level was an independent predictor of recurrence-free survival in M0 patients. We thought that the exact association between thrombus level and survival should be further studied. Second, we demonstrated that patients with higher Fuhrman grade, perinephric fat invasion, sarcomatoid differentiation or non-clear cell RCC had higher risk of PFS. These variables were also validated to be associated with overall survival or cancer-specific survival in previous studies (4, 5). Last, we confirmed the prognostic value of adjuvant therapy in PFS and the exact effect of adjuvant therapy on PFS need be evaluated in the future.

To the best of our knowledge, The PKUN model was the first nomogram predicting PFS in M0 RCC patients with venous thrombus. Abel et al. (18) once developed a nomogram predicting recurrence following surgery in M0 RCC patients with venous thrombus. Compared to their model, the PKUN model predicted not only the recurrence risk of M0 patients but also the risk of death. When recurrence or metastatic progression occurred, adjuvant therapy or salvage therapy was usually necessary. We believed that our nomogram could be used for enrollment assessment in future adjuvant therapy clinical trials. Actually, whether the application of PKUN model could lead to clinical benefit was a major concern. We performed the decision curve analysis and confirmed that PKUN could result in net benefit when the cutoff was 1% for 1-year PFS, 25% for 3-year PFS, and 40% for 5-year PFS.

From a clinical standpoint, the PKUN model improves the ability to identify patients with a higher risk of progression after surgery. Its implementation could help determine which kind of patients need active intervention and closer follow-up. However, the consistency between the predicted risk and the actual risk was another concern. In our study, we drew the calibration plots to assess the extent of overestimation or underestimation associated with PKUN use. The externally validated calibration curves of predicted probability against the observed PFS indicated excellent concordance. Besides that, the C-indexes showed that PKUN had good discrimination in clinical practice.

Distinct facets of our results deserve attention. First, our results showed that variables such as Fuhrman grade or pathological type were predictors of PFS, but not the thrombus level. These observations indicated that the inherent cancer characteristics were associated with disease progression. Second, we confirmed that patients receiving adjuvant therapy had a better PFS, though Gu et al. (19) reported in a prospective cohort study that adjuvant therapy showed no benefit in PFS for M0 RCC patients with venous thrombus. Last, although lymph node metastasis was not a predictive variable, we found that patients with lymph node metastasis had a worse PFS than N0M0 patients. The worse PFS for N1 patients indicated that rigorous follow-up and adjuvant therapy were necessary to improve the survival.

Our study has strengths in the prospective follow-up design and prospective data collection. In addition, the study period is from January 2014 to March 2021, and it can represent the current clinical practice, especially the comprehensive therapy based on surgical treatment. Despite several strengths, our study is not devoid of limitations. The first is its single-center experience nature, and it contains a relatively small number of patients. A multicenter prospective follow-up covering sufficient data is needed to better evaluate the PFS. Furthermore, a relatively shorter follow-up time limited the observation of progression events. This study would definitely benefit from a longer follow-up.

## Conclusion

We constructed and validated the PKUN model to predict the 1-year, 3-year, and 5-year PFS of M0 RCC patients with venous thrombus after surgery. The model can help counsel patients and identify patients who can benefit the most from surgery and develop the criteria for clinical trial enrollment.

## Data Availability Statement

The raw data supporting the conclusions of this article will be made available by the authors without undue reservation.

## Ethics Statement

This study receives ethics approval from Peking University Third Hospital Ethics Committee.

## Author Contributions

YZ: Data collection/project development/data analysis/article writing, XJT Project development/critical revision, HB Project development/data analysis/article writing, YY Project development, ZL Data collection/project development, SDZ Project development/critical revision, CL Project development/critical revision, LLM Project development/critical revision. All authors contributed to the article and approved the submitted version.

## Funding

This work was supported by the National Natural Science Foundation of China (81972381).

## Conflict of Interest

The authors declare that the research was conducted in the absence of any commercial or financial relationships that could be construed as a potential conflict of interest.

## Publisher’s Note

All claims expressed in this article are solely those of the authors and do not necessarily represent those of their affiliated organizations, or those of the publisher, the editors and the reviewers. Any product that may be evaluated in this article, or claim that may be made by its manufacturer, is not guaranteed or endorsed by the publisher.
